# High-velocity deformation of Al_**0**.3_CoCrFeNi high-entropy alloy: Remarkable resistance to shear failure

**DOI:** 10.1038/srep42742

**Published:** 2017-02-17

**Authors:** Z. Li, S. Zhao, H. Diao, P. K. Liaw, M. A. Meyers

**Affiliations:** 1 Materials Science and Engineering Program, University of California, San Diego, California 92093, USA; 2 Department of Materials Science and Engineering, The University of Tennessee, Knoxville 37996, USA

## Abstract

The mechanical behavior of a single phase (fcc) Al_0.3_CoCrFeNi high-entropy alloy (HEA) was studied in the low and high strain-rate regimes. The combination of multiple strengthening mechanisms such as solid solution hardening, forest dislocation hardening, as well as mechanical twinning leads to a high work hardening rate, which is significantly larger than that for Al and is retained in the dynamic regime. The resistance to shear localization was studied by dynamically-loading hat-shaped specimens to induce forced shear localization. However, no adiabatic shear band could be observed. It is therefore proposed that the excellent strain hardening ability gives rise to remarkable resistance to shear localization, which makes this material an excellent candidate for penetration protection applications such as armors.

Most commercial alloy systems are based on one principal element such as Fe, Al, Ni, with minor additions of other elements to improve properties. More recently, the high-entropy alloy design strategy has emerged, in which five or more elements are mixed in equiatomic or near equiatomic concentrations, such that the high configurational entropy 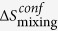 promotes solution formation^1^,^2^,^3^. The configurational entropy is often ignored in the phase prediction of conventional alloys, because they are based on one major element and their phases would have small mixing entropies. A randomly-equimolar mixed solid solution will have a high configurational entropy, which needs to be considered for the prediction of the equilibrium phases^4^. High-entropy alloys represent a new field of alloy development that focuses attention away from the corners of phase diagrams toward their centers.

Many high-entropy alloys have shown exceptional properties, including remarkable fracture toughness at low temperature^5^, high magnetization^6^, and resistance to fatigue^7^,^8^. More recently, Li *et al*.^9^ developed a non-equiatomic Fe_80−x_Mn_x_Co_10_Cr_10_ HEA system which exhibits both exceptional high strength and ductility at room temperature. The authors attribute the excellent performance to the versatile deformation mechanisms (including dislocation mediated plasticity, mechanical twinning, and stress induced phase transitions), producing a continuous and steady strain hardening.

The processes that occur when bodies are subjected to rapidly changing loads can differ significantly from those that occur under static or quasi-static situations^10^. When materials are subjected to large plastic deformation, the temperature increases because a large fraction of the plastic work is converted into heat. Localization of plastic flow can occur when the heating causes thermal softening that overcomes strain hardening and strain-rate hardening. Adiabatic shear bands (ASB) can act as precursors to brittle or ductile failure^11^. Thus, it is of great importance to study the dynamic response and microstructure evolution of high-entropy alloys.

## Results and Discussion

**Figure 1:**
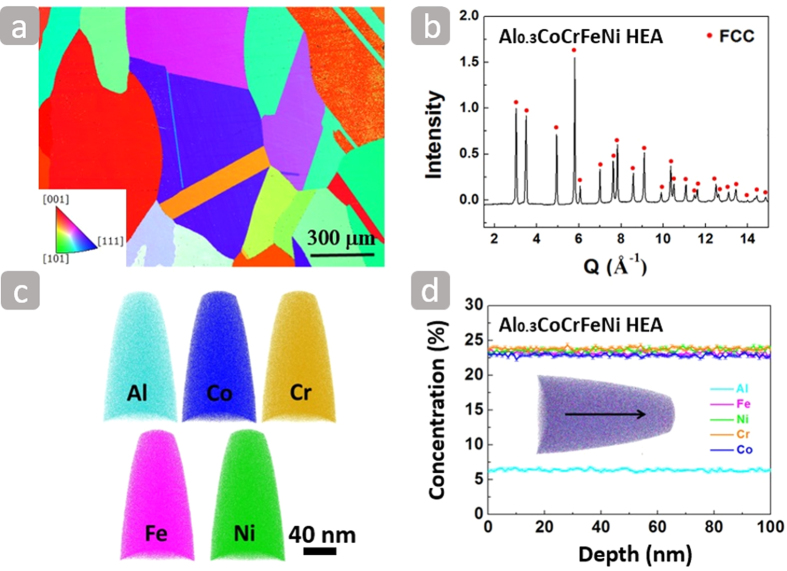
(**a**) Electron backscattered diffraction (EBSD)-inverse pole figure (IPF) showing the microstructure of the Al_0.3_CoCrFeNi high-entropy alloy with annealing twins. (**b**) Neutron-diffraction pattern^12^ of the Al_0.3_CoCrFeNi high-entropy alloy. (**c**) APT analysis showing the homogeneous distribution of Al, Co, Cr, Fe, and Ni elements. (**d**) APT results of one dimensional element concentration taken along the black arrow.

The alloy contains coarse grains with size ~500 μm and large annealing twins shown in Fig. 1(a). The single phase (fcc) Al_0.3_CoCrFeNi alloy, as demonstrated by neutron-diffraction pattern^12^ in Fig. 1(b), contains two elements (Cr and Fe) that crystallize with the body-centered cubic (bcc) structure, two elements (Ni and Al) as face-centered cubic (fcc) and one (Co) as hexagonal close-packed (hcp). This single phase fcc crystal structure is consistent with the XRD analysis of Al_x_CoCrFeNi (x in molar ratio, x = 0–2.0) high-entropy alloy^13^. The elemental maps of the Al_0.3_CoCrFeNi high-entropy alloy in Fig. 1(c) show that the distribution of Al, Co, Cr, Fe and Ni within the analyzed volume is homogeneous with no indication of segregation or clustering at the atomic scale. The atom probe tomography (APT) results of Fig. 1(d) show the concentrations of the different elements along with the black arrow, which represents the nominal alloy composition.

**Figure 2:**
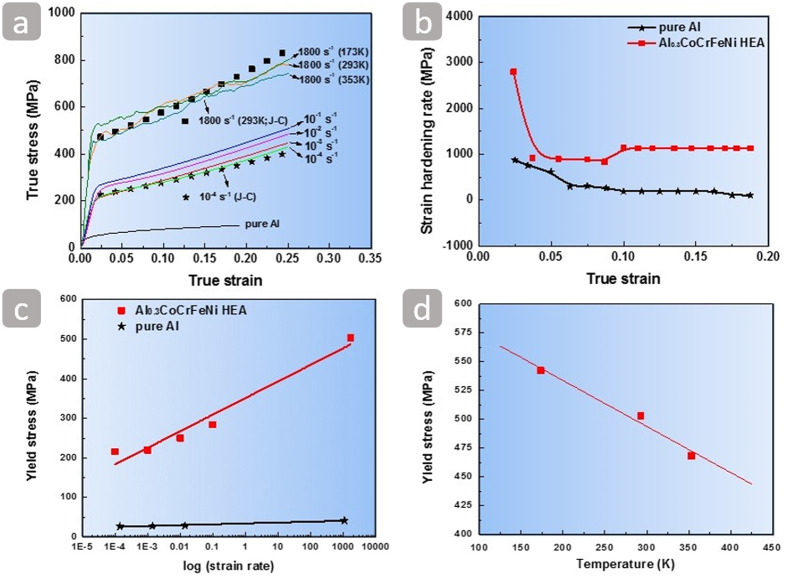
(**a**) True stress-true strain curves of the Al_0.3_CoCrFeNi high-entropy alloy at different strain rates (with Johnson-Cook model^23^ fitting curves) and true stress-true strain curve of the CG pure aluminum at the quasi-static 10^−2^ s^−1^. (**b)** Strain hardening rate as a function of true strain of the Al_0.3_CoCrFeNi high-entropy alloy and pure aluminum at the strain rate 10^−2^ s^−1^. (**c**) Yield stress as a function of log (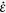). (**d**) Thermal softening of the Al_0.3_CoCrFeNi high-entropy alloy at 1800 s^−1^.

The compressive true stress-true strain curves at room temperature are plotted in Fig. 2(a). The Al_0.3_CoCrFeNi high-entropy alloy has a high strain hardening ability during plastic deformation both at quasi-static and high strain rates. The stress-strain curve of coarse-grained (CG) (grain size ~75 *μ*m) pure aluminum^14^ is also shown in Fig. 2(a) for comparison. Figure 2(b) shows changes of strain hardening rate θ (defined by 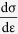) as a function of true strain for the alloy and CG pure Al at a strain rate of 10^–2^ s^−1^. The strain hardening rate of the alloy (above 1000 MPa) is significantly higher than that of CG pure Al. Figure 2(b) shows a negative-slope region followed by a positive-slope region. Such transition is due to a change in the deformation mechanism from dislocation slip-dominated plastic deformation to twinning-dominated plastic deformation. Work hardening suppresses both necking (in tension) and localization (in shear). Both phenomena are interconnected. Lu *et al*.^15^ showed that nanocrystalline copper exhibited much higher ductility when the grain boundaries are nanotwins. The cause of the high strain hardening in low-SFE fcc alloys, particularly high-Mn steels, has been widely discussed^16^. This high strain-hardening effect is due to the interstitial C atoms of C–Mn dipoles interacting strongly with dislocations or mechanical twins providing barriers to dislocation motion with increasing dislocation storage and thus decreasing their mean free path^17^. In the case of dynamic deformation, thermal softening plays an additional role. Xiong *et al*.^18^ reported a maximum temperature increase of 55 K for a twin-induced plastic TWIP steel deformed at a high rate of 2400 s^−1^. This temperature rise was not sufficient to form an adiabatic shear band. In single-phase high-entropy alloys, the lattice is locally distorted because of the occupation of the same crystallographic sites by atoms with different sizes. This local distortion effect is an essential strengthening mechanism^19^,^20^,^21^. The combination of several strengthening mechanisms such as solid solution hardening, forest dislocation hardening, as well as twinning can lead to an excellent work hardening ability. The yield stress *σ*_y_ of the alloy increases from 216 MPa to 503 MPa when the strain rate increases from 10^−4^ s^−1^ to 1.8 × 10^2^ s^−1^. The corresponding strain-rate sensitivity m (defined by 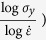 is about 0.053 as shown in Fig. 2(c). The strain-rate sensitivity of the pure Al is about 0.028, which is only half of that of the alloy^22^. In order to study the thermal softening, the dynamic response at the strain-rate 1800 s^−1^ and different temperatures was examined. The thermal softening parameter 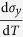 shown in Fig. 2(d) is ~−0.4 MPa/K.

The propensity to shear localization can be quantified from the constitutive response. The Johnson-Cook model is the most common phenomenological constitutive equation used to describe the plastic behavior of materials^10^. A modified Johnson-Cook constitutive equation^23^ is used here:


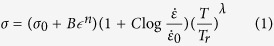


where *σ*_0_ is the yield stress, 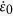 (10^−4^ s^−1^) is a reference strain rate and *T*_*r*_ (293 K) is a reference temperature. A, B, n, C and *λ* are experimentally determined parameters: *σ*_0_= 216 MPa, *B* = 1000 MPa, *C* = 0.145, *n* = 1.2, *λ* = −0.18, *T*_*r*_ = 293 K. The work of the deformation can be used to calculate an (adiabatic) temperature rise:


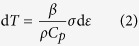


where ρ is the density and *C*_*p*_ is the specific heat capacity. The parameter β, which is the efficiency of the conversion of the strain energy into heat, is usually taken as 0.9. The density of the Al_0.3_CoCrFeNi high-entropy alloy was measured to be 7860 kg/m^3^. The specific heat *C*_*p*_ is approximated to be 460 J/kg·K by using a weight averaging method 
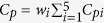
 (*w*_*i*_ is the weight percent and *C*_*pi*_ is the specific heat for each element of the alloy). A relationship between the temperature and plastic strain, at a fixed strain rate, is obtained by substituting Eq. (1) into Eq. (2):


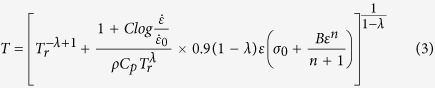


The temperature at an extreme high strain rate of 10^4^ s^−1^ and true strain of 0.5 is only 402 K, representing a temperature rise of 109 K.

Joseph *et al*.^24^ reported that both arc-melted and direct laser sintered Al_0.3_CoCrFeNi alloy samples (with FCC structure) exhibited a yield strength of 200 MPa, excellent strain hardening, and had tensile failure strain above 1. Li *et al*.^25^ discovered that tensile strength and elongation of the FCC Al_0.3_CoCrFeNi alloy increased with decreasing temperatures and reached 1010 MPa and 68% at 77 K. The Charpy impact energy of the Al_0.3_CoCrFeNi alloy was approximately 413 J at room temperature and 328 J at 77 K. This superior ductility and ultrahigh Charpy impact toughness of this alloy are the result of mechanial nanotwinning instead of planar slip dislocations^26^. This has been also observed by Gludovatz *et al*.^5^ in the CrMnFeCoNi high-entropy alloy. Deformation-induced nanotwinning delays the onset of necking instability (i.e., localized plastic deformation that can lead to premature failure) to higher strains.

**Figure 3:**
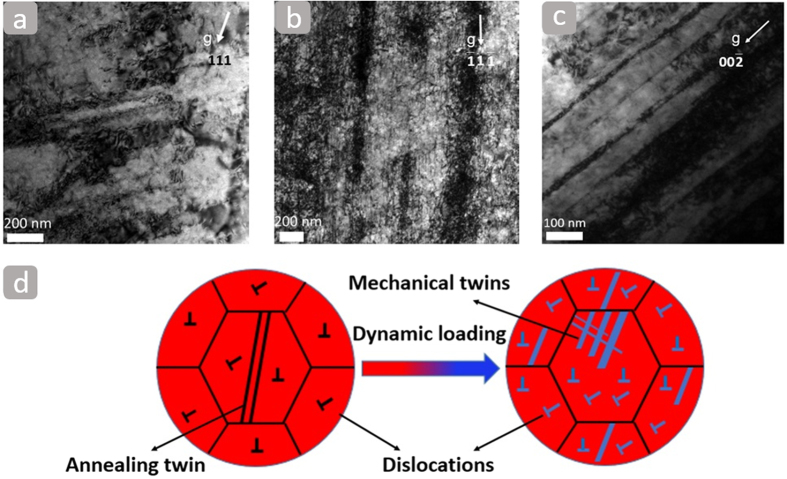
TEM bright-field images of the deformed samples at the strain rates of (**a**) 10^−4^ s^−1^; (**b**) and (**c**) 1800 s^−1^. (**d**) Schematic sketches showing the deformation mechanisms of the Al_0.3_CoCrFeNi high-entropy alloy under dynamic loading.

In order to understand the strain hardening mechanisms of the Al_0.3_CoCrFeNi high-entropy alloy, it is important to establish the microstructure evolution of deformed samples. This was examined at strain rates of 10^−4^ s^−1^and 1800 s^−1^ by transmission electron microscopy (TEM) and is shown in Fig. 3. Figure 3(a) shows tangles of randomly distributed dislocations in the sample deformed at 10^−4^ s^−1^, revealing that forest dislocations can lead to steady strain-hardening during plastic deformation. Ma *et al*.^27^ reported a superior high tensile elongation (of ~80%) for a monocrystalline Al_0.3_CoCrFeNi high-entropy alloy due to continuous dislocation motion. Figure 3(b) indicates that the high density of dislocations tends to align in one direction and entangle under dynamic loading. Such a high dislocation density configuration has been also observed for the laser-induced shock compression of monocrystalline copper^28^. Mechanical twins form in many metallic materials, especially those with low stacking-fault energy (SFE). Some fcc single phase materials with high SFE (such as Al and Ni) may not easily form twins except under extreme conditions, such as low temperatures and high strain rates^29^. Figure 3(c) shows that the parallel bands are mechanical twins, which suggests that mechanical twinning is an important plastic deformation mechanism in the Al_0.3_CoCrFeNi high-entropy alloy at high strain rates. Figure 3(d) summarizes the deformation mechanism of the Al_0.3_CoCrFeNi HEA under dynamic loading, indicating that the dislocation slip and twinning can lead to the excellent strain-hardening ability.

Proportionality between the twin-boundary energy and the stacking-fault energy (SFE) has been reported for most metals^30^. Some fcc single phase materials with high SFE^31^,^32^ (Al with SFE ≈ 86 mJ/m^2^ and Ni with SFE ≈ 120–130 mJ/m^2^) may not easily form twins except under extreme conditions, such as low temperatures and high strain rates. Kumar *et al*.^33^ reported low SFE (below 30 mJ/m^2^) of the Al_0.1_CoCrFeNi alloy. He attributed it to the larger atomic size difference between Al and other elements in the alloy. Low stacking-fault energy values (~30 mJ/m^2^) have been reported in NiFeCrCoMn alloy^34^. Hence, one would expect that addition of Al would cause a high lattice strain per atomic percent in CrFeCoNi as compared to Mn. Mishra *et al*.^35^ proposed that lattice strain in HEAs might play an important role in dislocation core energy which in turn will have a bearing on stacking-fault formation and its energy. The lattice strain in HEAs raises the base energy of the crystal and thereby reduces the additional energy required to nucleate dislocations and twins. Since Al_0.1_CoCrFeNi has ~1.2 wt.% of Al, it is assumed that Al addition might have caused a decrease in SFE from ~30 mJ/m^2^ to a lower value. Thus, it is proposed that the SFE of Al_0.3_CoCrFeNi is lower than that of the Al_0.1_CoCrFeNi alloy, leading to the formation of profuse mechanical twins in high strain-rate deformation.

Shear localization has been found to be an important and sometimes dominant deformation and fracture mode in metals, granular ceramics, polymers, and metallic glasses at high strains and strain rates^10^. For metals, thermal softening is the first stage of this process, leading to processes of dynamic recovery and recrystallization with associated drops in the flow stress. Calculations by Meyers *et al*.^36^ revealed that the break-up of the elongated sub-grains and diffusive rotation of the grain boundaries can occur during the deformation process. The complex inter-relationships between stress, stress state, strain, strain rate and temperature, have been used for pursuing a better design of materials with the objective of postponing and even avoiding localized shear deformation.

An adiabatic shear band can form when the material starts to “soften”. The condition for instability is 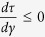, is obtained from the general function *τ* = *f(γ*,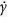,*T*):


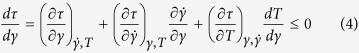


The normal stress and strain shown in Equation (1) at a constant strain rate of 10^4^ s^−1^ can be converted to the corresponding shear stress and shear strain^37^ by: 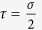 and 
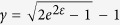
. For a shear strain of 1.1, at constant 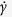 and *T*, 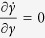, the strain hardening parameter is ~440 MPa, and the thermal softening parameter is ~−0.2 MPa/K. Consequently, 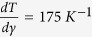. Neglecting the second term in Equation (4):


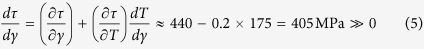


Thus, shear instability is not predicted from the constitutive response of this alloy.

**Figure 4:**
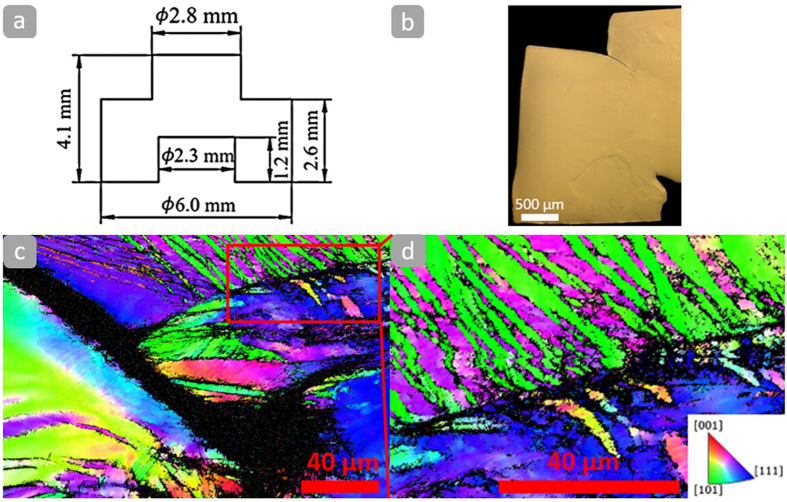
(**a**) Dimension of the hat-shaped sample. (**b**) Optical microscopy image of the deformed hat-shaped sample. (**c**) IPF mapping near the deformation tip. (**d**) IPF mapping showing deformation twinning in the vicinity of the tip.

The resistance of the Al_0.3_CoCrFeNi alloy to shear localization was then experimentally studied by dynamically-loading a hat-shaped sample to induce forced shear localization. The dimension of the hat-shaped sample is shown in Fig. 4(a). Figure 4(b) shows the optical image of the deformed hat-shaped sample. The electron backscatter diffraction (EBSD)-inverse pole figure (IPF) mapping in Fig. 4(c) indicates that the hat-shaped sample was highly deformed under high strain rates but not fractured. The formation of mechanical twins can be observed in Fig. 4(d) near the deformation tip. No observable shear band can be identified in the hat-shaped sample at an imposed shear strain of ~1.1. The microstructure is severely deformed in the “forced” shear region. Xu *et al*.^38^ studied shear localization and recrystallization in dynamic deformation of a CG single fcc 8090 Al-Li alloy and observed a recrystallized equiaxed structure with an average grain size of ~0.2 *μ*m. Dynamic recrystallization, which has been widely observed inside adiabatic shear bands^10^, is absent in the shear region of the hat-shape sample, indicating the alloy continues to harden under dynamic loading instead of softening by recrystallization. Jiao *et al*.^39^ reported pile-ups around the indents in the Al_0.3_CoCrFeNi high-entropy alloy and observed a highly localized severe plastic deformation under the nanoindentation. However, they did not mention shear bands. Indeed, pile-ups are a natural result of indentations because of the volume constraining requirement. Tang *et al*.^40^ found that the grain size of the Al_0.3_CoCrFeNi high-entropy alloy decreased severely from ~350 *μ*m to nanocrystalline size due to dynamic recrystallization via high-pressure torsion processing method. These observations indicate that dynamic recrystallization of this alloy can happen under severe plastic deformation. However, adiabatic shear localization of this alloy can only be activated by higher strains and temperatures than the ones imposed by our experiments. The enlarged Fig. 4(d) with a ~36% twin area fraction and a ~2.4 *μ*m average twin thickness confirms that their formation can play an important role on high strain-rate deformation. Consequently, the dislocation slip and twin-twin reaction can lead to the excellent strain-hardening ability; this, in turn, results in the its extraordinary resistance to shear localization.

**Figure 5:**
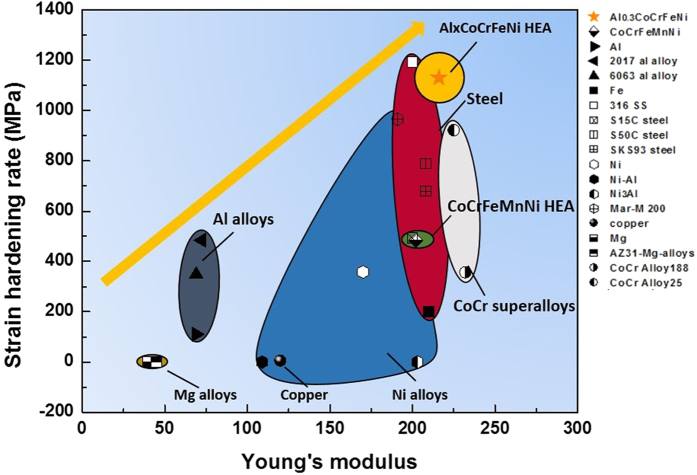
Comparison of strain-hardening ability of Al_0.3_CoCrFeNi HEA with other structural materials. For consistency, strain-hardening rates were calculated at a fixed true strain of 0.2 (in compression) for all materials except for pure Mg which usually fractured at this point.

In summary, we investigated the mechanical response and microstructural evolution of a high-entropy alloy with emphasis on the high strain-rate regime. The yield stress σ_y_ increases from 216 MPa to 503 MPa with increasing strain rate from 10^−4^ s^−1^ to 1800 s^−1^, showing a significant strain-rate sensitivity of 0.053. The strain hardening rate of the alloy is also significantly higher than a CG pure Al and retained in the dynamic regime. The TEM images and EBSD analysis of dislocations and mechanical twinning reveal that (1) the high strain-hardening ability, enabled by solid solution hardening, forest dislocation hardening and twinning hardening, (2) the high strain-rate sensitivity and (3) modest thermal softening give rise to the high resistance to shear localization. Figure 5 shows the excellent performance of the Al_0.3_CoCrFeNi HEA studied here, in comparison with other alloys. Our results suggest that the Al_0.3_CoCrFeNi high-entropy alloy maintains the remarkable mechanical properties at high strain rates, rendering it to be of great potential for impact-protection (ballistic) applications.

## Methods

### Sample Processing

The Al_0.3_CoCrFeNi high-entropy alloy was fabricated by vacuum-induction melting with the Al, Co, Cr, Fe and Ni elements to cast a plate of ~127 mm × 305 mm × 19 mm. Then the plate underwent the hot-isostatic-pressing (HIP) at 1,204 °C and 103 MPa for 4 hours to reduce defects formed during the casting and cooling processes. Samples were cut from the center of the bulk materials and underwent homogenization at 1,200 °C for 2 hours, followed by water quench.

### Mechanical testing

The quasi-static compression tests of cylinders were performed in an Instron Universal Testing Machine with the dimensions of 4 mm in diameter and 6 mm in length. Dynamic compression tests were performed using a Split Hopkinson Pressure Bar (SHPB). The SHPB was used to test the dynamic response and shear deformation using two kinds of specimens: cylinders and hat-shaped samples, respectively. The cylinders for dynamic tests have a length of 6 mm and a diameter of 6 mm and were placed in a sealed temperature controlled chamber. Temperature inside the chamber was recorded by thermocouples. Hat-shaped specimens were used to generate high localized shear strain and induce forced shear localization.

### Microstructure characterization

Neutron-diffraction experiments were performed at room temperature on the Nanoscale-ordered Materials Diffractometer (NOMAD) at the Spallation Neutron Source (SNS) located at Oak Ridge National Laboratory. This aerodynamic levitator provides a containerless environment, in which HEA samples (2 mm diameter spheres) are suspended above a conical nozzle by flowing argon gas. Samples for the electron backscattered scanning diffraction (EBSD) observation were prepared by standard mechanical grinding and polishing with the colloidal Al_2_O_3_. The sharp-tip specimens for atom-probe tomography (APT) were prepared in a FEI Nova 200. The APT experiments were conducted using a CAMECA local electrode atom probe (LEAP), 4000X HR, equipped with an energy-compensated reflection lens. The APT measurements were performed in voltage mode at the temperature of 50 K, pulse frequency of 200 kHz, and pulse fraction of 20%, respectively. At least 5 million ions were collected for each sample to ensure adequate data statistics. The datasets were reconstructed and analyzed using the IVAS 3.6.8 software (CAMECA Instruments). To prepare TEM specimens, deformed samples were cut to slices of *ϕ*3 × 1 mm using a linear cutting machine and then slices were mechanically polished to a thickness of 70 *μ*m. Foils with a diameter of 3 mm for the TEM examination were prepared by TenuPol-3 with an etching solution of 30% volume nitric acid and 70% volume methanol. TEM samples were characterized by transmission electron microscopy (TEM) using a model Tecnai G^2^ Polara FEI electron microscope operating at 200 kV.

## Additional Information

**How to cite this article**: Li, Z. *et al*. High-velocity deformation of Al_0.3_CoCrFeNi high-entropy alloy: Remarkable resistance to shear failure. *Sci. Rep.* **7**, 42742; doi: 10.1038/srep42742 (2017).

**Publisher's note:** Springer Nature remains neutral with regard to jurisdictional claims in published maps and institutional affiliations.
